# Underlying Mechanisms and Neurorehabilitation of Gait after Stroke

**DOI:** 10.3390/brainsci12091251

**Published:** 2022-09-16

**Authors:** Janis J. Daly, Svetlana Pundik, Jessica P. McCabe

**Affiliations:** 1 Brain Plasticity and NeuroRecovery Laboratory, Cleveland VA Medical Center, Cleveland, OH 44106, USA; 2 Department of Neurology, Case Western Reserve University, Cleveland, OH 44016, USA; 3 Brain Rehabilitation Research Center, Malcom Randall VA Medical Center, Gainesville, FL 32608, USA; 4 Department of Physical Therapy, College of Public Health and Health Professions, University of Florida, Gainesville, FL 32608, USA

The title of this *Special Issue* is: “Underlying Mechanisms and Neurorehabilitation of Gait after Stroke”. There have been numerous excellent studies focusing on gait neurorehabilitation after stroke. However, persistent gait deficits continue to result in debilitating disability and poor quality of life after stroke. Traditionally, gait training has focused on peripherally administered treatments such as limb exercise, balance training, robotics, functional electrical stimulation, treadmill training, and aerobics. The mechanisms of the neural drive for normal gait coordination are complex and currently poorly understood, as are the mechanisms of neural drive required for recovery after stroke. New discoveries of central nervous system mechanisms and function are relevant to the development of more beneficial gait training methods.

Therefore, the topic of this issue is important, because to effectively move the field of gait neurorehabilitation forward for stroke survivors, there is need for more sophisticated methods, especially in the realm of neural control. To that end, it is important to incorporate current state-of-the-art and emerging neural function research into the task of developing precise neural function outcome measures and interventions. The opinion paper in this *Issue* by McCabe et al. [1] lays out the logic and evidence for this direction of inquiry. Indeed, there are numerous nodes of neural control of gait, spanning the central and peripheral nervous systems, including cortex, basal ganglia, thalamus, brain stem, spinal cord, and peripheral nerve and muscle. Each of these nodes of control has a unique contribution to the motor control of normal gait and post-stroke gait dyscoordination and balance deficit. Understanding the functionality of these neural regions is needed to direct the field of gait neurorehabilitation.

Important emerging work on neural mechanisms of gait control has been published, and examples are provided in this *Issue* in the opinion paper by McCabe et al. [1]. In addition, in this *Issue*, a case report by McCabe et al. [2] provides quantitative evidence that traditional strength training may be necessary for restoring gait movement components, but strength training is not sufficient alone; rather, coordination training is necessary for those stroke survivors for whom dyscoordination is present. Furthermore, a paper in this *Issue* by Litinas et al. [3] provides quantitative biomechanical mechanism evidence that gait rehabilitation in stroke survivors should include coordination training for activating the coordinated movements of the normal gait pattern, and also that gait neurorehabilitation should include the motor control involved in selectively de-activating muscles, so that the precisely coordinated biomechanical forces of normal gait can be engaged for optimal gait energy cost and ease of movement. The paper by Komiya et al. [4] describes a novel balance training protocol that was designed to target the mechanisms of somatosensory and vestibular systems of balance after stroke; their interesting results provide justification for increasing the treatment duration and intensity of their protocol. Salameh et al. [5] developed and tested a novel protocol designed to target the mechanism of brain excitability through non-invasive direct current stimulation (tDCS); their protocol included applying this non-invasive brain stimulation during stance phase training, and they framed the intervention within a motor learning paradigm. Their results showed that the brain stimulation was, in fact, received in the M1 (lower limb) region during balance training. The test subjects in this short brain stimulation treatment protocol responded with equal or greater improvement in functional gait measures than other studies with longer, peripherally directed treatment duration. The preliminary results justify the continued study of that protocol, potentially in a randomized, controlled trial.

There are two papers and a commentary in this *Issue* addressing the topic of gait measurement. First, Chow and Stokic [6] present evidence from a group of stroke survivors that significant change in gait speed and other gait characteristics can be measured from shortly after the stroke to 6 months post-stroke. However, from 6 to 12 months post-stroke, there was no change. These findings are important in illustrating the importance of selecting chronic stroke survivors (≥6 months post-stroke) to test the efficacy of new interventions in the absence of the confound of endogenous recovery that occurs during the first 6 months post-stroke. At the same time, the opinion paper in this *Issue* [1] calls for measures that are more sophisticated than the compensatory measure of gait speed, and describes some of the inadequacies of using gait speed as a single measure or as a primary measure with no accompanying explanatory impairment measures such as gait coordination or balance control [1]. In fact, the commentary in this Issue (by Daly [7]) n association with the Chow and Stokic paper [6]) utilizes the published data in the Chow paper to illustrate potential errors in conclusions that can occur with the use of a gait speed measure in the absence of measures of gait coordination or balance control. To address this issue for those who do not have access to the technology required for gait coordination measures of gait kinematics or kinetics, a paper in this *Issue* by Daly et al. [8] offers a report on a measure of gait coordination that can be used in any clinical or research laboratory. This is available in several languages, and is in use internationally [8].

There are three papers in this *Issue* that target important aspects of the peripherally directed neurorehabilitation of gait after stroke. The first, by Komiya et al. [4], provides a report on response to balance training. The second, by McCabe et al. [2], provides evidence for the importance of coordination training. The third paper, by Awosika et al. [9], describes results in response to a backward training protocol, which in effect is a type of coordination training. A fourth paper in this Issue, by Salameh et al. [5], answers the call for treatment that targets the neural drive of the gait pattern. This paper describes the successful development and testing of a protocol targeted to improve the stance phase of gait and which uses a sophisticated method of brain neural stimulation while walking (administered within the framework of evidence-based motor learning methods [5]).

Taken together, these representative papers are evidence that it is now critical for gait interventions after stroke to be elevated to the level of targeting neural control in treatment and measuring neural change associated with the recovery of gait coordination. Important measurement methods will include quantifying neural structures, pathways, and mechanisms. The existing literature supports the need for this inflexion point in neurorehabilitation gait training after stroke, and calls for more precise methods of neural measurement and intervention. It is our hope that the collection of papers in this *Issue* will assist in justifying future work and will spark discussion and creative future approaches to elucidating the underlying mechanisms of normal gait coordination and the recovery of gait coordination for the benefit of stroke survivors.
